# The effect of negative, single and multi‐organism positive cultures on outcomes following PCNL

**DOI:** 10.1002/bco2.70150

**Published:** 2026-01-01

**Authors:** Katya Hanessian, Ali Albaghli, Ruben Crew, Grant Sajdak, Ala'a Farkouh, Sikai Song, Daniel Jhang, Zham Okhunov, D. Duane Baldwin

**Affiliations:** ^1^ Department of Urology Loma Linda University Health Loma Linda California USA

**Keywords:** fever, infection, nephrolithiasis, percutaneous nephrolithotomy, sepsis

## Abstract

**Objective:**

This study aims to explore risk factors related to positive single and multi‐organism stone cultures and their association with postoperative complications in patients undergoing percutaneous nephrolithotomy (PCNL).

**Subjects/Patients and Methods:**

A retrospective review was performed on 293 PCNL patients with stone cultures at a single academic institution between January 2017 and March 2023. Data collection encompassed demographics, comorbidities, operative details and postoperative outcomes. Chi‐square and ANOVA with Tukey B post hoc tests were employed. Multivariable logistic regression identified independent outcomes. Significance was set at *p* < 0.05.

**Results:**

Positive stone cultures were seen in 56% of patients and cultures with multiple organisms were seen in 25% of patients. Female sex (*p* = 0.007), preoperative nephrostomy tubes (*p* < 0.001) and longer surgical durations (*p* < 0.001) were more likely to have positive cultures. Significant associations were observed between positive cultures and postoperative fever (*p* = 0.007), readmissions (*p* = 0.020), stone recurrence (*p* = 0.002) and multidrug resistance (*p* = 0.016) with no difference between single‐ and multi‐organism culture groups. Positive cultures were independently associated with higher odds of readmission (OR = 4.31; *p* = 0.03) and stone recurrence (OR = 2.89; *p* = 0.005). Additionally, calcium phosphate and struvite stones were associated with positive cultures (*p* < 0.001).

**Conclusion:**

Positive stone cultures (single or multi‐organism) predicted adverse postoperative outcomes including fever, readmission and recurrence. Patients with multi‐organism stone cultures were more likely to have stone recurrences within 6 months, suggesting the need for closer follow‐up and more comprehensive antibiotic therapy. These findings emphasize the role of stone culture status in guiding risk stratification and tailored prophylactic strategies, particularly in patients with multi‐organism stone cultures who have multidrug resistance.

## 1 INTRODUCTION

Kidney stones affect 10% of the US population, with a rising prevalence in the last decade.^1^ With 77% of renal calculi harbouring bacteria,^2^ infectious complications like fever, sepsis and systemic inflammatory response syndrome (SIRS) are common after percutaneous nephrolithotomy (PCNL) cases. Following PCNL, 17.6% of patients develop SIRS, while 4.5% experience sepsis, contributing to greater morbidity and mortality.^3^ While it is established that factors such as female sex, hydronephrosis, preoperative nephrostomy tubes, large or complex stone burdens and comorbidities like diabetes and obesity increase the risk of postoperative sepsis following PCNL,^4^, ^5^, ^6^ multidrug resistant organisms (MDROs) and biofilm formation exacerbate these risks despite use of appropriate prophylactic antibiotics.^7^, ^8^, ^9^, ^10^ No studies have comprehensively explored how these risk factors vary across negative, single‐organism and multi‐organism stone cultures, particularly in the context of emerging antimicrobial resistance patterns.

Sepsis from stone‐related infections remains a severe, potentially fatal complication.^11^ Despite the widespread use of preoperative urine cultures and antibiotics, preventing infectious complications post‐PCNL is challenging, as these measures often fail to predict stone‐specific infections.^12^ Intraoperative stone cultures offer more reliable guidance for targeted antibiotic therapy, yet the evolving microbial landscape, including an increase in Gram‐positive organisms, fungi, and MRDOs, complicates treatment.^4^, ^12^, ^13^, ^14^, ^15^ The rise of extended‐spectrum beta‐lactamase (ESBL)‐producing bacteria and carbapenem‐resistant Enterobacteriaceae (CRE) has reduced the efficacy of standard perioperative antibiotics.^16^, ^17^ Furthermore, delays in stone culture results (typically 2–3 days) mean that surgeons do not adjust perioperative antibiotics based on these results in up to 64% of cases,^2^ which is complicated by the increasing prevalence of antibiotic‐resistant pathogens.

These challenges highlight the importance of investigating the risk factors associated with positive stone cultures and their impact on postoperative complications and outcomes. This study aims to investigate these risk factors and predictive variables associated with positive stone cultures and their impact on postoperative outcomes.

## 2 SUBJECTS/PATIENTS AND METHODS

After obtaining approval from the Institutional Review Board #5230274, a retrospective review was conducted on patients who underwent PCNL at our institution from January 2017 to March 2023. Patients who had intraoperative stone cultures were included in the analysis. Children under 18 and patients who did not obtain intraoperative stone cultures during PCNL were excluded from the study.

Data collection included patient demographics like age, sex and body mass index (BMI). Preoperative parameters and stone characteristics that were gathered included American Society of Anesthesiology (ASA) score, stone type, organism profile, presence of hydronephrosis, preoperative indwelling tubes and preoperative serum white blood cell count (WBC). Outcomes included total surgery duration, stone analysis, intraoperative antibiotics, postoperative intensive care unit (ICU) admission, postoperative sepsis, postoperative fever, readmission within 30 days, stone recurrence and stone recurrence time. Stone recurrence was defined as stone formation that required surgery for removal but did not include second look procedures.

The data was analysed with SPSS version 27 (IBM, Armonk, NY) and version 4.1.0 of R (R Foundation for Statistical Computing, Vienna, Austria). Univariate analysis was used to determine clinical associations comparing factors correlated with positive cultures using chi‐squared analysis for categorical variables and ANOVA test for continuous variables. Tukey B was performed for post hoc test. Multivariable regression modelling identified factors independently associated with outcomes. Each statistical test was two‐sided. Statistical significance was defined as a *p*‐value of <0.05.

## 3 RESULTS

### 3.1 Patient characteristics and risk factors for positive stone cultures

**FIGURE 1:**
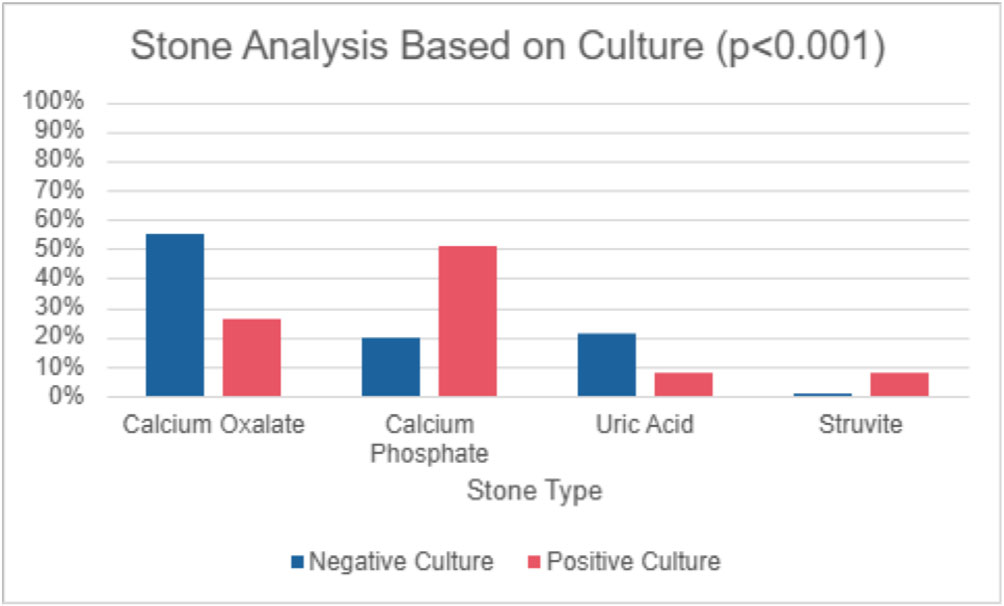
Stone analysis based on culture.

A total of 293 patients who underwent PCNL were included in the study. Of these, 197 patients had stone cultures with 56% (*n* = 111) being positive (Table 1) and 25% (*n* = 49) being positive for multiple organisms (Table 2). Ninety‐six patients did not obtain stone cultures and were excluded from the study. The median age was 57 years for patients with positive stone cultures and 63 years for those with negative cultures (*p* = 0.13). The most common organism genus in our cohort was *Enterococci*. Median BMI was 30 kg/m^2^ for both groups (*p* = 0.9). Positive stone cultures are associated with female sex (*p* = 0.007), preoperative Foley catheters (*p* = 0.036), ureteral stents (*p* = 0.010) and nephrostomy tubes (*p* < 0.001). Those with multi‐organism stone cultures had a higher proportion of patients with an ASA score of 3–4 and with indwelling catheters compared to those with single‐organism or negative cultures (*p* < 0.05 for all). Calcium phosphate (51% vs. 20%) and struvite stones (8% vs. 1%) were more likely to have positive versus negative cultures (*p* < 0.001, Figure 1). No significant associations were observed between groups based on the presence of staghorn stones (*p* = 0.4), presence of hydronephrosis (*p* = 1.0) and serum WBC count (*p* = 0.9).

**TABLE 1:** Patient demographics, characteristics and outcomes.

Parameter	Stone negative (*n* = 86)	Stone positive (*n* = 111)	*p*‐value
Age*	63 (50, 71)	57 (42, 67)	0.13
**Sex (*n*, %)**			**0.007**
**Male**	**50 (58%)**	**43 (39%)**	
**Female**	**36 (42%)**	**68 (61%)**	
BMI (kg/m^2^)*	30 (26, 35)	30 (25, 35)	0.9
ASA			0.55
1	1 (1%)	2 (2%)	
2	43 (50%)	38 (34%)	
3+	42 (49%)	71 (64%)	
Staghorn stone (*n*, %)	69 (80%)	94 (85%)	0.515
Presence of hydronephrosis (*n*, %)	41 (48%)	53 (48%)	0.957
**Operative time (min)** *	**156 (133, 195)**	**182 (152, 246)**	**<0.001**
**Preoperative catheter (*n*, %)**	**3 (4%)**	**13 (12%)**	**0.036**
**Preoperative ureteral stent (*n*, %)**	**6 (7%)**	**22 (20%)**	**0.010**
**Preoperative nephrostomy (*n*, %)**	**6 (7%)**	**29 (26%)**	**<0.001**
Preoperative serum WBC Count (K/uL)*	7.26 (6.40, 8.74)	7.74 (5.80, 9.57)	0.9
Postoperative ICU admission (*n*, %)	0 (0%)	1 (0.9%)	>0.9
Postoperative sepsis (*n*, %)	0 (0%)	4 (4%)	0.13
**Postoperative fever (*n*, %)**	**1 (1%)**	**12 (11%)**	**0.007**
**Readmission (*n*, %)**	**8 (9%)**	**24 (22%)**	**0.020**
**Stone recurrence (*n*, %)**	**6 (7%)**	**26 (23%)**	**0.002**

* Continuous variables were reported by median values and interquartile ranges (IQR).

**TABLE 2:** Patient characteristics and outcomes based on multi‐organism, single‐organism and negative stone culture.

Parameter	Negative culture (*n* = 86)	Positive single culture (*n* = 62)	Positive multiple culture (*n* = 49)	*p*‐value
Age (years)*	58.4	53.6	57.2	0.15
**Sex**				**0.026**
**Male (*n*, %)**	**50 (58%)**	**24 (39%)**	**19 (39%)**	
**Female (*n*, %)**	**36 (42%)**	**38 (61%)**	**30 (61%)**	
BMI (kg/m^2^)*	30.4	32.4	29.2	0.1
**ASA Score (*n*, %)**				**0.03**
**1–2**	**44 (51%)**	**26 (42%)**	**14 (29%)**	
**3–4**	**42 (49%)**	**36 (58%)**	**35 (71%)**	
**Pre‐op Foley (*n*, %)**	**3 (4%)**	**3 (5%)**	**10 (20%)**	**0.001**
**Pre‐op nephrostomy (*n*, %)**	**6 (7%)**	**9 (15%)**	**20 (41%)**	**<0.001**
**Pre‐op ureteral stent (*n*, %)**	**6 (7%)**	**12 (19%)**	**10 (20%)**	**0.037**
Operative time (min)*	**167.4**	**209.9**	**183.5**	**<0.001**
Readmission within 30 days (*n*, %)	8 (9%)	14 (23%)	10 (20%)	0.06
**Post‐op fever (*n*, %)**	**1 (1%)**	**7 (11%)**	**5 (10%)**	**0.025**
Post‐op sepsis (*n*, %)	0 (0%)	2 (2%)	2 (4%)	0.196
Post‐op ICU (*n*, %)	0 (0%)	0 (0%)	1 (2%)	0.22
**Stone recurrence (*n*, %)**	**6 (7%)**	**16 (26%)**	**10 (20%)**	**0.006**
**Recurrence time (*n*, %)**				**0.005**
**<6 months**	**5 (5%)**	**7 (11%)**	**7 (14%)**	
**6–12 months**	**0 (0%)**	**3 (5%)**	**3 (6%)**	
**>12 months**	**1 (1%)**	**6 (10%)**	**0 (0%)**	
**Multidrug Resistance**	‐‐‐‐	**33 (67%)**	**43 (87%)**	**0.016**
**Stone culture directed intraoperative antibiotics**	‐‐‐‐	**47 (56%)**	**37 (44%)**	**0.001**

### 3.2 Culture concordance and perioperative data

In the face of negative urine cultures, patients were placed empirically on cefazolin and gentamicin unless patients had renal insufficiency in which case non‐nephrotoxic antibiotics were selected. When cultures were positive, patients were treated with culture‐directed antibiotics. Of the 111 patients with positive stone cultures, intraoperative antibiotics appropriately covered micro‐organisms in 84/111 (75.7%) stone cultures and did not cover organisms in 27/111 (24.3%) stone cultures (Table 3). Of the 111 positive stone cultures, 17 (15.3%) grew fungi. In a comparison of cultures based on the concordance of organisms between preoperative urine and stone cultures, patients with concordant cultures were less likely to be readmitted within 30 days (*p* = 0.043) and less likely to develop postoperative fever (*p* = 0.028; Table 4). However, no differences were observed in sepsis, ICU admission or stone recurrence between concordant and non‐concordant cultures.

**TABLE 3:** Univariate analysis observing intraoperative coverage of organisms in stone cultures.

Outcome	Abx covered (84)	Abx not covered (27)	*p*‐value
Post‐op WBC	11.7	12.8	0.23
Presence of fungi	**7 (8%)**	**10 (37%)**	**<0.001**
Presence of *Pseudomonas*	13 (15%)	5 (19%)	0.71
Multiple organisms	37 (44%)	12 (44%)	0.97
Readmission in 30 days	15 (17%)	9 (33%)	0.09
Post‐op fever	7 (8%)	5 (19%)	0.14
Post‐op sepsis	3 (4%)	1 (4%)	0.97
Post‐op ICU	0 (0%)	1 (4%)	0.08
Stone recurrence	18 (21%)	8 (30%)	0.38

**TABLE 4:** Univariate analysis comparing concordance of organisms between preoperative urine and stone cultures.

Variable	Non‐concordant (*n* = 19)	Concordant (*n* = 52)	*p*‐value
**30‐day readmission**	**7 (37%)**	**7 (13%)**	**0.043**
**Postop fever**	**5 (26%)**	**3 (5.8%)**	**0.028**
Postop sepsis	2 (11%)	1 (1.9%)	0.2
Stone recurrence	6 (32%)	11 (21%)	0.4

### 3.3 Patient outcomes

Positive stone cultures were significantly associated with longer operative times (*p* < 0.001), postoperative fever (*p* = 0.007), readmission within 30 days (*p* = 0.020) and stone recurrence (*p* = 0.002). Patients with multi‐organism stone cultures were most likely to have stone recurrences within 6 months (*p* = 0.005). Positive stone culture was an independent risk factor for both stone recurrence (odds ratio [OR] = 2.89, 95% confidence interval [CI]: 1.10 to 8.43; *p* = 0.03, Table 5) and 30‐day readmissions (OR = 4.31, 95% CI: 1.5 to 14.1; *p* = 0.005). Male patients were also more likely to be readmitted within 30 days (OR = 4.59, 95% CI: 1.83 to 12.84; *p* < 0.001). While positive stone cultures were more commonly observed in patients with postoperative sepsis (4% vs. 0%, *p* = 0.13) and those requiring postoperative intensive care unit (ICU) admission (0.9% vs. 0%, *p* > 0.9), these associations did not reach statistical significance. There were no differences in postoperative complications between single‐ and multi‐organism stone cultures (all *p* > 0.05); however, multi‐organism cultures showed a trend toward higher rates of multidrug resistance (*p* = 0.016).

**TABLE 5:** Multivariable regression of stone recurrence and 30‐day readmission.

Parameter	Stone recurrence			30‐day readmission		
	OR	Cl	*p*‐value	OR	Cl	*p*‐value
**Positive stone culture**	**2.89**	**1.10–8.43**	**0.03**	**4.31**	**1.52–14.11**	**0.01**
Age (years)*	0.98	0.94–1.01	0.22	1.00	0.95–1.04	0.83
**Male sex**	0.80	0.33–1.90	0.61	**4.59**	**1.83–12.84**	**0.00**
BMI (kg/m^2^)*	0.97	0.92–1.01	0.16	0.99	0.94–1.05	0.79
Staghorn	1.01	0.34–3.49	0.98	0.45	0.15–1.42	0.17
Hydronephrosis	1.41	0.62–3.26	0.41	0.90	0.38–2.11	0.82
Operative time (min)*	1.00	1.00–1.01	0.17	1.00	1.00–1.01	0.14
Preoperative catheter	2.76	0.78–9.54	0.11	1.02	0.19–4.17	0.98
Preoperative stent	0.78	0.21–2.37	0.67	1.34	0.39–4.20	0.62
Preoperative nephrostomy tube	0.76	0.26–2.02	0.59	1.27	0.41–3.78	0.67
Preoperative serum WBC	1.11	0.98–1.26	0.09	1.11	0.98–1.28	0.09

## 4 DISCUSSION

This retrospective cohort study provides a comprehensive analysis of the risk factors, patient characteristics and postoperative outcomes associated with positive stone cultures in patients undergoing PCNL over a 6‐year period. Our study demonstrated that patients with positive cultures, in both single‐ and multi‐organism groups, were more likely to be female, had a higher prevalence of preoperative Foley catheters, ureteral stents and nephrostomy tubes.^4^, ^5^ Notably, positive cultures were linked to higher rates of postoperative fever, readmission within 30 days and stone recurrence, with positive stone cultures independently associated with increased odds of stone recurrence (OR = 2.89) and 30‐day readmission (OR = 4.31). Positive cultures were also associated with calcium phosphate and struvite stones. Multi‐organism stone cultures were most likely to have MDROs present in their stones.

Historically, urologists have primarily relied on urine cultures to guide antibiotic treatment to target active infection or prophylaxis following PCNL.^18^, ^19^ While this is an accepted clinical practice, it may not accurately reflect the infectious burden of the stone itself. The core of the stone harbours distinct and potentially more aggressive bacterial flora,^20^ often shielded from antibiotic exposure before surgical intervention. Recent studies suggest that biofilm growth within stones contributes to persistent infections and antibiotic resistance, particularly with MDROs such as ESBL‐producing *Escherichia coli* and CRE.^16^, ^17^ During lithotripsy, fragmentation releases these bacteria into the collecting system, increasing the risk of systemic infection. This risk is further exacerbated in prolonged surgeries or when urothelial integrity is compromised, coupled with high intrarenal pressure due to instrumentation and manipulation during PCNL.

Despite the well‐established value of stone cultures in predicting postoperative infectious complications, their clinical utility remains largely overlooked due to the inherent delay in obtaining results. In fact, a study by Osman et al. suggests use of stone cultures to guide stone management did not change antibiotic regimens in most patients.^14^ However, we hypothesize that we do not see a significant difference because providers do not adjust antibiotic treatment due to the delay in obtaining stone culture results. Typically, stone culture results take 3–5 days to process, by which time most patients have already developed fevers or been discharged, forcing clinicians to rely on preoperative urine cultures for treatment. Up to 77% of kidney stones harbour bacteria,^2^ which is consistent with the 56% of patients in our study who had positive stone cultures. The significant association between positive stone cultures and infection‐related complications, along with increased surgical complexity, underscores the limitations of urine cultures alone in predicting postoperative infections. These findings support the routine adjustment of postoperative management based on intraoperative stone cultures for those with risk factors to guide more targeted antibiotic therapy, reduce infection‐related complications and rise of MDROs and improve patient outcomes. Our results also emphasize timely management of infection post‐PCNL, which may warrant alternatives to detect pathogens in renal calculi such as polymerase chain reaction or reveal biofilm formation such as scanning electron microscopy.^21^, ^22^

Medical and surgical management of struvite stones pose a challenge to the field of endourology due to their proliferative growth in the renal collecting system and association with harboring bacteria. Extended operative times in patients with positive cultures suggest the technical difficulty of managing infected, complex stones, such as struvite types, as these tend to grow rapidly and occupy the entire collecting system.^12^ Struvite stones were prevalent in positive stone cultures, as they are known to harbor urease‐splitting pathogens,^13^ but calcium phosphate stones had the highest prevalence in multi‐ and single‐organism stone cultures. This may be due to biofilm formation which serves as a nidus for calcium phosphate crystal aggregation,^23^, ^24^ or urease‐splitting uropathogens alkalinizing urine, which can be a substrate for calcium phosphate formation.^25^, ^26^ Currently, urologists give prophylactic antibiotics to select postoperative patients with struvite stones, but it is not standard practice to treat patients with calcium phosphate stones with prophylactic antibiotics.^13^, ^27^ Clinicians can target patients with calcium phosphate stones preoperatively by implementing interventions to prevent alkalinization and bacterial colonization using measures such as dietary and pharmacologic measures to acidify urine or inhibiting urease activity through antibiotics and vitamin C.^28^, ^29^ Based on our findings, we recommend that the management of calcium phosphate stones should include serial urine cultures with targeted antibiotic treatment for detected infections as well as imaging such as renal ultrasound during follow‐up visits to assess for recurrence. Given the higher recurrence risk associated with infection‐related stones, this proactive approach may facilitate early detection and intervention, improving patient outcomes.

The identification of preoperative indwelling devices as significant risk factors for infected stones is consistent with current literature.^4^ These devices, commonly employed to manage obstruction or infection prior to PCNL, may inadvertently facilitate bacterial colonization of stones, particularly by *Enterococcus* species, which are associated with poorer clinical outcomes.^15^, ^29^, ^30^ The predominance of *Enterococcus*, a Gram‐positive organism often resistant to standard perioperative antibiotics like cephalosporins, highlights the necessity for tailored antimicrobial strategies. Optimizing infection control measures may involve minimizing device dwell times and modifying empirical antibiotic regimens to incorporate targeted agents like vancomycin or ampicillin to reduce the risk of postoperative complications. Additionally, our findings also support that integration of stone cultures results in postoperative antibiotic selection, in particular for high‐risk patients with persistent signs and symptoms of postoperative infection, to improve patient outcomes by enabling more precise pathogen‐direct therapy.

Positive cultures have traditionally been associated with sepsis and postoperative complications. In the study by Falaharkar et al., 17% of patients in the United States developed fever after PCNL.^3^ Our study corroborates with these findings as 11% of patients with positive stone cultures developed fevers postoperatively in our cohort. While previous studies have linked positive cultures to complications like sepsis and postoperative infections,^14^ this study did not find a significant association with postoperative sepsis or ICU admissions, possibly due to aggressive antibiotic treatment in these populations. However, the high prevalence of postoperative fever in patients with positive cultures suggests that infection‐related morbidity remains a concern. The lack of significant differences between multi‐organism and single‐organism cultures for sepsis or readmission, despite higher fever and recurrence rates, indicates that the sheer presence of infection, rather than the number of organisms, may drive deleterious postoperative infectious complications. Patients with multi‐organism stone cultures were more likely to have sooner recurrence and grow MDRO, which are associated with poor postoperative outcomes.^7^, ^8^, ^9^ These findings suggest that biofilm presence on stones or other virulence factors from MDROs may be a nidus for stone recurrence. The use of stone culture directed, targeted antibiotics based on stone culture findings, rather than broad‐spectrum antibiotics, supports more effective treatment, promotes antibiotic stewardship and may prevent stone recurrence.

Understanding risks of individual organisms may help urologists recognize when to treat with more intensive therapy. With the growing threat of MDROs, exacerbated by delayed culture results (2–3 days) and frequent antibiotic adjustments (up to 64% of cases), there is a strong rationale for advocating stone culture‐directed regimens to improve patient outcomes and mitigate resistance. This approach can benefit patients who exhibit signs of persistent infection despite empiric therapy or those at high risk for MDROs, such as patients with indwelling devices or recurrent UTIs.

Our study has several limitations that must be considered in addition to its retrospective nature. It was performed at a single academic institution, which may limit the generalizability of the findings to other populations. Additionally, the study involved multiple surgeons and operative techniques evolved over the study's multiyear span, potentially introducing variability in outcomes. Nevertheless, these results provide a compelling case for clinicians to leverage stone cultures for personalized patient management, while calling for multicentre studies to refine protocols and mitigate MDRO risks, which are steps that could potentially reduce post‐PCNL complications and improve patient prognosis.

## 5 CONCLUSION

The study highlights the importance of identifying risk factors for stone infection, optimizing preoperative and postoperative care and utilizing stone cultures to guide stone treatment. Calcium phosphate stones demonstrated an association with bacterial colonization, suggesting previously underrecognized infection‐related mechanisms of stone formation and recurrence. Positive stone cultures independently predicted readmission and stone recurrence; multiple organisms present on cultures are associated with multidrug‐resistant infections, which may drive adverse outcomes. Patients with multi‐organism stone cultures were more likely to have stone recurrences within 6 months, suggesting the need for closer follow up and more comprehensive antibiotic therapy.

## AUTHOR CONTRIBUTIONS

*Conceptualisation*: Katya Hanessian, Ali Albaghli, Zham Okhunov, and D. Duane Baldwin. *Methodology*: Ali Albaghli, Zham Okhunov, Ala'a Farkouh, Daniel Jhang and D. Duane Baldwin. *Investigation*: Katya Hanessian, Ruben Crew, Grant Sajdak, Ala'a Farkouh and Sikai Song. *Statistical analysis*: Ala'a Farkouh and Daniel Jhang. *Writing – original draft*: Katya Hanessian, Ruben Crew, Ala'a Farkouh and Daniel Jhang. *Writing – review and editing*: Ali Albaghli, Ala'a Farkouh, Zham Okhunov and D. Duane Baldwin. *Supervision*: Zham Okhunov and D. Duane Baldwin.

## CONFLICT OF INTEREST STATEMENT

No conflicts of interest to disclose.
